# One-Week Scutellar Somatic Embryogenesis in the Monocot *Brachypodium distachyon*

**DOI:** 10.3390/plants11081068

**Published:** 2022-04-14

**Authors:** Houssein Wehbi, Camille Soulhat, Halima Morin, Abdelhafid Bendahmane, Pierre Hilson, Oumaya Bouchabké-Coussa

**Affiliations:** 1 Université Paris-Saclay, INRAE, AgroParisTech, Institut Jean-Pierre Bourgin (IJPB), 78000 Versailles, France; h.wehbi.91@gmail.com (H.W.); camille.soulhat@inrae.fr (C.S.); oumaya.bouchabke@inrae.fr (O.B.-C.); 2 Université Paris-Saclay, CNRS, INRAE, Univ Evry, Institute of Plant Sciences Paris-Saclay (IPS2), 91190 Gif-sur-Yvette, France; halima.morin@inrae.fr (H.M.); abdelhafid.bendahmane@inrae.fr (A.B.)

**Keywords:** *Brachypodium distachyon*, development, monocot, regeneration, somatic embryogenesis, zygotic embryogenesis

## Abstract

Plant somatic embryogenesis (SE) is a natural process of vegetative propagation. It can be induced in tissue cultures to investigate developmental transitions, to create transgenic or edited lines, or to multiply valuable crops. We studied the induction of SE in the scutellum of monocots with *Brachypodium distachyon* as a model system. Towards the in-depth analysis of SE initiation, we determined the earliest stages at which somatic scutellar cells acquired an embryogenic fate, then switched to a morphogenetic mode in a regeneration sequence involving treatments with exogenous hormones: first an auxin (2,4-D) then a cytokinin (kinetin). Our observations indicated that secondary somatic embryos could already develop in the proliferative calli derived from immature zygotic embryo tissues within one week from the start of in vitro culture. Cell states and tissue identity were deduced from detailed histological examination, and in situ hybridization was performed to map the expression of key developmental genes. The fast SE induction method we describe here facilitates the mechanistic study of the processes involved and may significantly shorten the production of transgenic or gene-edited plants.

## 1. Introduction

Somatic embryogenesis (SE) is an excellent illustration of plant developmental plasticity. Through this process, which takes place in the absence of sexual reproduction, somatic cells change fate, acquire the ability to divide, and yield embryonic structures. In vitro culture methods based on SE are routinely applied for the genetic transformation, genome editing, or clonal propagation of a wide range of plant species [1,2,3]. This developmental pathway also offers an interesting model to study the molecular, regulatory, and morphogenetic events associated with the initiation of embryogenesis, as the cellular and molecular processes involved are still only partially understood.

For example, the acquisition of embryogenic competence, a crucial initial step of SE, will be better comprehended by closely tracking the morphological and histological events that occur during the somatic–embryogenic transition. Several studies focusing on calli cultured in vitro have illustrated the histological differences between either somatic and embryogenic tissues [4,5] or pluripotent meristematic cells and totipotent embryogenic cells [6]. However, most of the published plant SE studies do not provide a comprehensive analysis of the process. They often explore late SE stages, past the initial cell fate transitions, and most reports focusing on early stages characterize the cellular events [6,7] or the subtending molecular shifts [8,9,10] occurring in the embryogenic tissues separately.

The induction of SE in plant tissues is influenced by several factors. The composition of the culture medium is key, as exogenous molecules trigger the genetic reprogramming that leads to cell (de)differentiation and the acquisition of the embryogenic state. Among the medium components, plant growth regulators are by definition crucial since they control plant development and embryogenesis [11,12]. Among such regulators, auxins and cytokinins, which orchestrate cell division and (de)differentiation, are critical factors to the plant embryogenic response [13,14,15].

Furthermore, each stage of the SE process, from the specification of the embryonic cell fate to the formation of the seedling, is also controlled by genetic, biochemical, and physiological factors that drive the transition from the somatic to embryogenic state; molecular aspects of these transitions have been revealed by studying genes specifically expressed during SE [16,17]. These genes highlight several predominant functional categories as they code for proteins that contain extracellular domains, are components of the cell wall, or are involved in cell division and cell proliferation, or enzymatic post-translational modifications [18,19,20,21,22,23,24].

*WUSCHEL* and *WUSCHEL-related homeobox (WUS/WOX)* genes, which code for homeodomain transcription factors, are key players and major markers of particular cells during SE. The ectopic expression of the *Arabidopsis thaliana WUS* gene promotes the vegetative-to-embryogenic transition and the formation of embryos after only a small number of cell divisions [25]. It can also redirect root cells towards at least two different developmental pathways: shoot organogenesis and SE [26]. The overexpression of *WUS* also promotes SE and induces organogenesis in *Gossypium hirsutum* [27], increases somatic embryo production four-fold in *Coffea canephora* [28], increases callogenesis and embryogenesis, and similarly enhances the embryogenic potential of hairy root fragments in *Medicago truncatula* [29]. *WOX13* is a key regulator of callus formation and tissue repair after injury in *A. thaliana* [30]. In the gymnosperm *Picea abies*, *WOX2* and *WOX8/9* are transcribed at higher levels in the early stages of somatic and zygotic embryogenesis [31]. The expression of *WOX11* and *WOX5* in the founder cell layer and middle cell layer of the callus, respectively, indicates that these two genes may play a role in the mechanism of pluripotency acquisition during callus formation in *A. thaliana* [17].

The *BABY BOOM* (*BBM*) gene, a member of the *AINTEGUMENTA-like/PLETHORA* (*AIL/PLT*) family, encodes an APETALA2/ETHYLENE RESPONSE FACTOR (AP2/ERF) DNA-binding transcription factor that activates a complex of gene networks linked to different developmental processes, including embryogenesis, through the regulation of cell totipotency [23,32,33,34,35]. *BBM* was first identified as a marker gene expressed in microspore-derived *Brassica napus* embryos [32]. When ectopically expressed, the *BBM* gene induces embryo development from the cotyledon, leaf, and shoot apex in *A. thaliana* and from the hypocotyls of germinated seedlings in Brassica, without the application of exogenous hormones [32,36,37].

As a grass and monocot model, *Brachypodium distachyon* (hereafter referred to as *Bd*) is a useful species to investigate developmental mechanisms that take place in plant tissue cultures. For example, *Bd* studies have provided novel insight into the biology of the cell wall [38], zygote division, gene expression patterns, and auxin fluxes during early embryogenesis, which differ between monocots and eudicots [39].

The microscopic analyses described herein show that secondary embryos are formed within immature *Bd* scutellum in as little as 6 days from the start of in vitro tissue culture. As previously reported [4,5,24,40,41,42], callogenic growth induced by the synthetic auxin 2,4-D initiates in a narrow zone of the scutellar epithelium, closest to the middle part of the immature zygotic embryo (izEmb), where the scutellum vasculature connects with the main axis of the embryo proper linking the shoot and root apices. After three days of treatment with auxin, clusters of cells with embryogenic potential can be readily observed in these emerging meristematic regions. These scutellar clusters are competent for rapid morphogenesis, as they yield fully structured secondary embryos after only three additional days of treatment with a cytokinin (kinetin).

Our observations have practical implications as they suggest that protocols for the creation of transgenic or edited *Bd* lines may be significantly shortened in time. These results also bear on our understanding of the mechanisms driving plant cell dedifferentiation and the onset of embryogenesis. The developmental transitions involved occur in just a few cell-division cycles in explants that have relatively simple structures and are thus easier to characterize molecularly and cytologically.

The aim of this study was to streamline the induction of somatic embryogenesis in a model monocotyledonous species as a prerequisite to the molecular and genetic dissection of the mechanisms involved. Our goals were to determine how fast secondary embryos can be produced and which cell types or tissues are required for the process.

## 2. Results and Discussion

**Figure 1:**
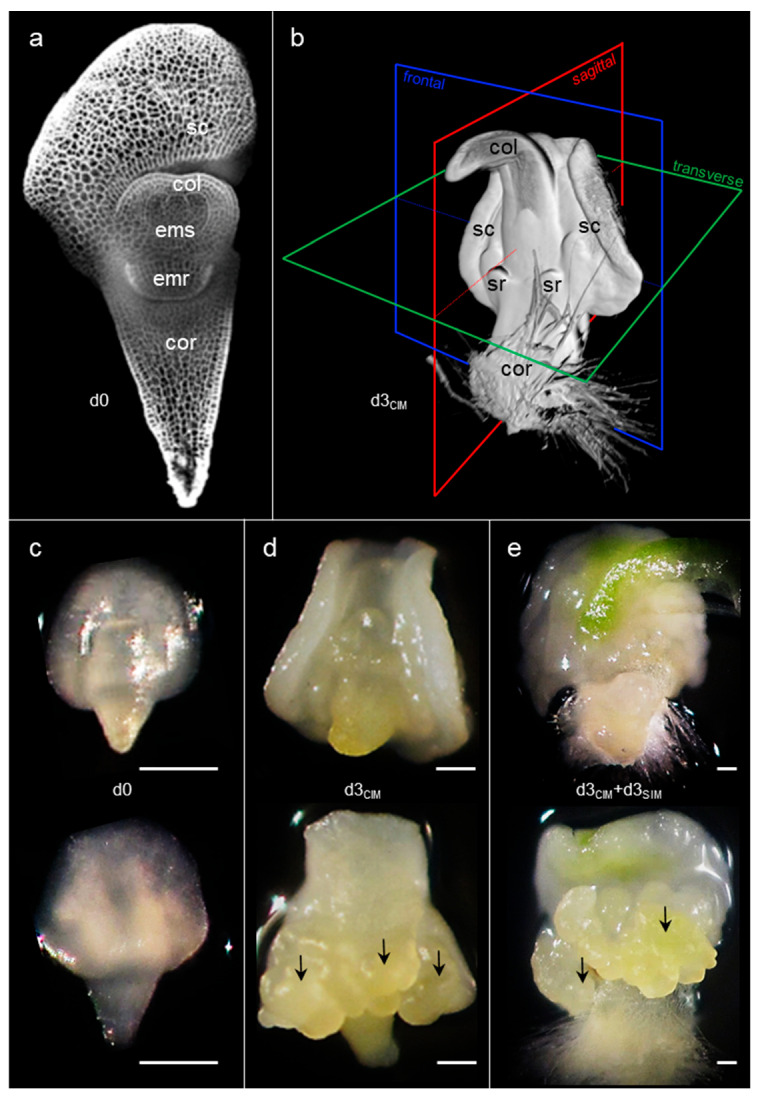
Structure of the *Bd* izEmb explant. Nine days after pollination (d0), zygotic immature embryos collected for in vitro tissue culture were already well established, as shown in a frontal confocal section of a propidium iodide-stained explant (**a**) or imaged on CIM (**c**). After three days of culture on CIM (d3_CIM_), the scutellum was substantially enlarged and formed two wings that wrapped the embryo proper (graphic representation, (**b**)). At this stage, proliferative bulges emerged (marked with black arrows) from the scutellar epithelium at the back of the explant, in a ring positioned around its middle section (**d**). After an additional three days of inoculation on SIM (d3_CIM_ + d3_SIM_), the proliferative bulges grew further, resulting in the production of callus tissues (**e**). Panels (**c**–**e**) provide front (top) and back (bottom) views of the same explant. col—coleoptile; cor—coleorhiza; emr—embryonic root; ems—embryonic shoot; sc—scutellum; sr—seminal root. Scale bar: 250 μm.

Nine days after pollination, the zygotic *Bd* embryo is already a complex 500 μm long organism, with multiple components. At the apices of its main axis, the embryonic shoot and root are surrounded by the coleoptile and coleorhiza, respectively, which form protective sheaths around the growing organs (Figure 1a,c). The embryo proper is connected to the scutellum, affixed in the seed against the triploid endosperm, through parenchymal and vascular tissues. At this stage, *Bd* izEmbs can be collected from flowers and induced to form secondary somatic embryos by treatment with exogenous phytohormones on gelled media.

For our descriptive study of the explants, we adopted the following 3D positioning convention: logically, the shoot apex points up and the root apex down, while the scutellum is positioned at the back of the explant and the two seminal roots are located in front (Figure 1b).

In tissue culture, the embryo proper grew according to a program very similar to that observed in the seed [38] within the time frame and under the conditions considered in this study. In stark contrast, parts of the scutellum developed rapidly in the presence of exogenous auxin (2,4-D, 11.3 µM; CIM) and, within three days, large proliferative bulges appeared at the back of the enlarged scutellum (Figure 1b,d). Following treatment with a cytokinin (kinetin, 0.9 µM; SIM), neo-formed secondary somatic embryos emerged from these bulges after a few additional days (Figure 1e).

To track the cellular events leading to the formation of new somatic embryos, *Bd* izEmb explants were collected at successive stages, fixed, and embedded in paraffin. Tissular details were analyzed in 8 μm thick sections stained with the periodic acid Schiff (PAS) reagent, which colors polysaccharides (pink), and naphtol blue black (NBB) dye, which marks proteins (blue) and nuclei (dark blue). The combined staining revealed cell boundaries, starch granules, xylem thickening, and division events that were important to our detailed analysis of tissue and organ development.

### 2.1. Cell Proliferation Induced by Exogenous Auxin Initiates Early in Restricted Peripheral Regions of the Scutellum

**Figure 2:**
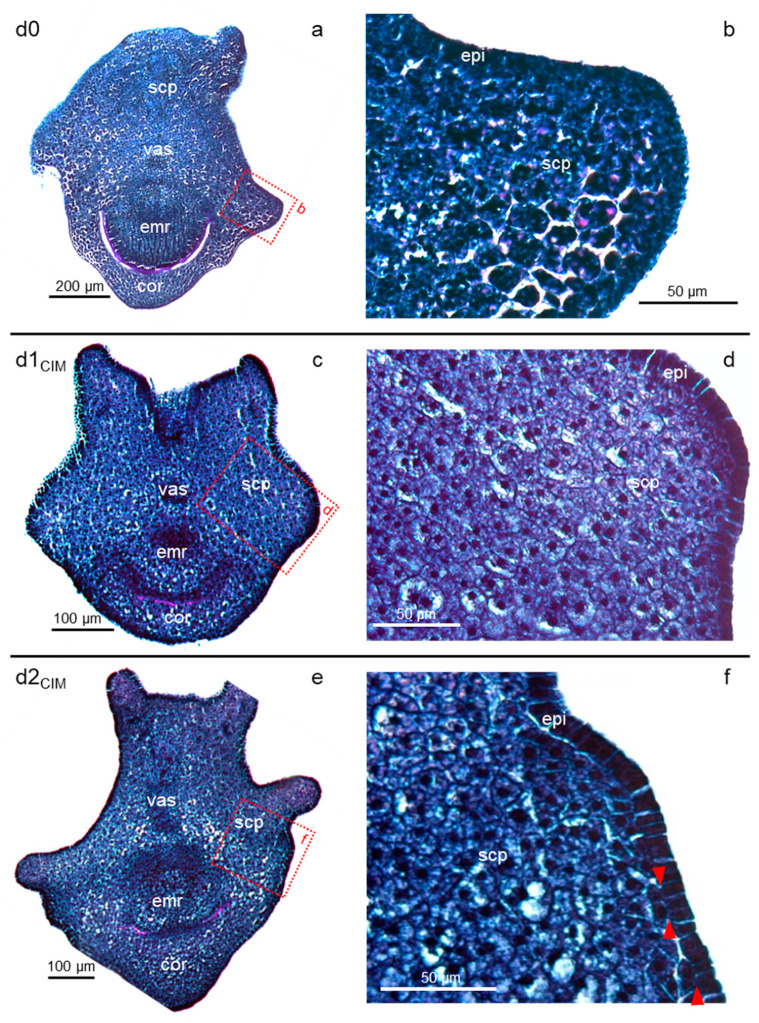
Initiation of callogenesis. Panels show frontal to oblique PAS-NBB-stained sections of izEmb explants at the beginning of induction with 2,4-D (**a**,**b**), one day after induction (**c**,**d**), and two days after induction (**e**,**f**). Cell morphology changes were observed after one day. The first epidermal cells divided in the periclinal orientation after two days (red triangles). Dotted red boxes indicate close-up views of the cross-sections. cor—coleorhiza; emr—embryonic root; epi—scutellum epidermis; scp—scutellum parenchyma; vas—scutellum vasculature.

For *B. distachyon*, as for cereals, regeneration methods based on immature zygotic embryos can be initiated with auxin-induced callogenesis (on CIM). In the first hours of in vitro tissue culture, all embryonic *Bd* tissues retained the same organization as in the in planta izEmb, with compact and darkly stained cells (Figure 2a,b). However, after only one day (d1_CIM_), some parenchyma cells of the scutellum appeared clearer than their neighboring epithelial cells, likely because the inner cells contained larger vacuoles, with a central nucleus and pink dots corresponding to starch granules (Figure 2c,d). The granules are most probably present at the earlier stage (d0) and become easier to discern as cells grow in size. In sections perpendicular to the surface of the explant, the scutellum epithelial cells were organized as stacked anticlinal rectangles, i.e., their longer axis was perpendicular to the outer surface of the organ (Figure 2d). In an arc positioned at the bottom of the dorsal scutellum, bordering the upper part of the coleorhiza, some cell divisions occurred after two days of tissue culture (d2_CIM_) in the epidermis or in the cells adjacent to it (Figure 2e,f). The resulting disorganized pattern was restricted to small patches with a width of four to five cells at most.

**Figure 3:**
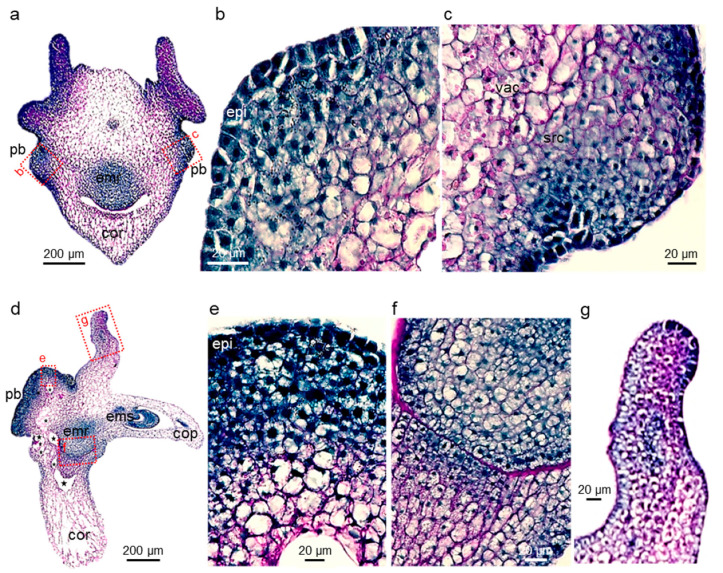
Formation of embryogenic cell clusters. Panels show frontal (**a**–**c**) or sagittal (**d**–**g**) PAS-NBB-stained sections of d3_CIM_ izEmb explants. Dividing cells resulted in the formation of bulges in a narrow region of the scutellum epidermis. From the outside in, the proliferative bulges were composed of dark dividing cells, then mid-size starch-rich cells, and finally large vacuolated cells with purple boundaries. Tissues in the vicinity of the bulges were ruptured and contained alveoli (dark stars). Dotted red boxes indicate close-up views of the cross-section. cor—coleorhiza; emr—embryonic root; epi—scutellum epidermis; pb—proliferative bulge; scp—scutellum parenchyma; src—starch-rich cells; vac—vacuolated cells; vas—scutellum vasculature.

One day later (d3_CIM_), the scutellum pattern initiated earlier became manifest (Figure 3). Most of the inner scutellum parenchyma cells were bigger (up to 40 μm in diameter) than any other cell types in the explant, with their growth being driven by an increase in the vacuolar space that now occupied the majority of the cell volume. Consequently, the scutellum was enlarged (Figure 3a) compared to its shape in the immature embryos collected nine days after pollination (Figure 2a). Most of the inner scutellum parenchyma cells were pink colored because of their starch granules, and possibly because additional polysaccharides were deposited in the cell wall, while their cytoplasm and nucleus were pushed to the outer edge of these large cells (Figure 3b,c). At this stage (d3_CIM_), some epithelial and sub-epithelial cells proliferated actively with division planes in various orientations. They were clustered in the areas where the very first epithelial cell divisions were observed and formed bulges that pushed the explant surface outward.

However, not all portions of the scutellum contained reactive tissues, as illustrated in a near sagittal cross-section of a separate explant (Figure 3d–g). Massive proliferation was observed only at the back of the izEmb explant, corresponding to the scutellum epidermal region near the back coleorhiza. The enlargement of tissues in that restricted portion of the izEmb caused major mechanical strains that bent the embryo axis and tore the parenchyma of the scutellum and coleorhiza in multiple locations (Figure 3d). The structure of small, blue-stained cells found in the scutellar proliferative bulges (Figure 3c–d) were similar to that of other mitotically active cells in the developing embryo axis (Figure 3f). In other portions of the scutellum, no or very few cell divisions were observed in the epidermis and none seemed to occur in the subtending cell layers. For example, in the thinner wings of the scutellum, the epidermal cells were immediately adjacent to compact starch granule-rich parenchymal cells, and these tissues did not proliferate (see upper protrusion; Figure 3g). The comparison between proliferative and non-proliferative tissues indicates that dividing (blue) cells did not contain packed starch granules and cells that contained them (purple) did not divide.

### 2.2. Cell Division and Developmental Genes Mark Scutellar Embryogenic Tissues

**Figure 4:**
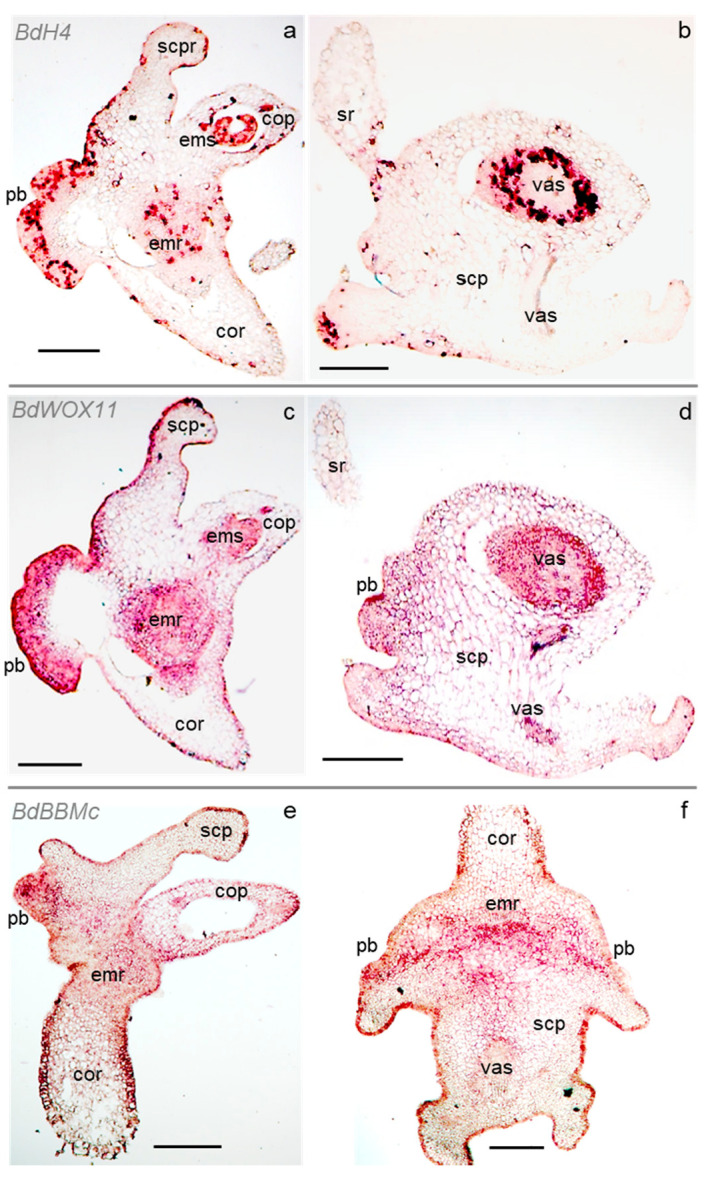
Expression of developmental genes in embryogenic tissues. Panels show RNA in situ hybridization of antisense RNA probes in cross-sections of d3_CIM_ izEmb explants, in the sagittal (**a**,**c**,**e**) or frontal (**b**,**d**,**f**) orientation. Detection of the transcript yields a ruby red signal, while some probes are associated with a brown background (compare with sense RNA probe controls in Supplementary Figure S1). cop—coleoptile; cor—coleorhiza; emr—embryonic root; ems—embryonic shoot; pb—proliferative bulge; scp—scutellum parenchyma; sr—seminal root; vas—scutellum vasculature. Scale bar: 200 μm.

To locate potential meristematic regions in the scutellum, we studied the expression pattern of selected genes in izEmb cross-sections through ISH (Supplementary Methods). Fixed d3_CIM_ explants were characterized for this purpose because our data suggested that an important switch in cell identity occurred at this stage, leading to the formation of new meristems (Figure 4 and Supplementary Figure S1).

First, the precise localization of *histone H4* (*BdH4*) transcripts was determined to have a detailed map of the cell divisions induced by exogenous auxin in the scutellum. *H4* nucleosome proteins are encoded by a highly conserved gene family, and *H4* transcription is a reliable indicator of mitotic activity as it peaks sharply in the early S-phase of the cell cycle [43]. As illustrated in d3_CIM_ izEmb cross-sections, the *BdH4* probe specifically marked several domains with multiple mitotically active cells after three days of exogenous auxin induction (Figure 4a). In the embryo proper, cell divisions take place in several files of the embryonic root, in all the first leaf tissues, and in the coleoptile vascular bundles. In contrast, virtually no division could be detected in the parenchyma of the connective tissues nor in that of the coleorhiza (Figure 4a). In agreement with our observations of PAS-NBB-stained cross-sections, the most proliferative cells were located at the base of the back scutellum epidermis, corresponding to the rapidly growing bulges. Interestingly, a sharp boundary separated the smaller peripheral proliferative cells from the inner larger cells of the scutellum parenchyma that do not divide, indicating that the bulges were only derived from the outermost cell layers. In the depth of the scutellum, the only *BdH4*-marked cells were restricted to short files associated with vascular bundles (Figure 4b). In the same section, *BdH4* transcripts marked cells at the base of the seminal roots. The single-layer epidermis that wrapped around the zygotic structures (including the back of the scutellum, also called the epithelium on the endosperm side) presented a complex distribution of cell divisions: at this stage, multiple divisions had already formed localized proliferative bulges, while very few epidermal cells had divided in the upper portion of the scutellum, in the coleoptile, or in the coleorhiza (Figure 4a).

The *WUSCHEL* (*WUS*) and *WUSCHEL-related homeobox* (*WOX*) genes code for plant transcription factors that control growth and development. Specific members of the *WOX* gene family are known regulators of meristem functions and stem cell homeostasis. Their ectopic overexpression has also been shown to induce SE in both dicot and monocot species [34]. Among 13 *WOX* genes we identified in the *Bd* genome (Supplementary Figure S2 and Supplementary Table S2), two were found to be transcribed in d3_CIM_ izEmb explants by RT-PCR analysis: *BdWOX11* (Bradi1g63680) and *BdWOX13a* (Bradi2g53390). Their expression pattern was further characterized in ISH experiments with gene-specific probes (Supplementary Methods; Supplementary Table S3). While *BdWOX13a* expression could not be detected, *BdWOX11* transcripts marked multiple tissues in izEmb cross-sections. In the embryo proper, the *BdWOX11* antisense probe labeled cells in the coleoptile vasculature, the first leaf, and the embryonic shoot, as well as in the embryonic root and the coleorhiza (Figure 4c). In the scutellum, the *BdWOX11* probe labeled cells in the central vascular bundle and its ramifications, the newly formed proliferative bulges, and certain segments of the epidermis, but not in the parenchyma (Figure 4c,d). Thus, the *BdH4* and *BdWOX11* expression domains largely overlap but are not identical. *BdWOX11* is expressed at high levels within dividing tissues of the embryo proper, as well as in proliferative portions of the scutellum that form upon 2,4-D induction. This observation suggests that cells in the latter tissues have acquired a novel fate.

To investigate a potential cell identity shift, we analyzed the expression of members of the *BABY BOOM* (*BbBBM*) gene family. The BBM AP2 transcription factors control pluripotency and are associated with the initiation of plant embryogenesis [23,32,34]. The rice *OsBBM1*, *OsBBM2*, and *OsBBM3* genes are expressed in gametes and zygotes where they function redundantly, as only triple loss-of-function mutants show strong early embryogenesis defects. In particular, *OsBBM1* expressed initially in the sperm cell triggers zygotic pluripotency after fertilization and its ectopic expression induces parthenogenesis in the egg cell [44]. We identified *BdBBM* genes closely homologous to these three rice genes (Bradi1g64240, Bradi2g57747, Bradi3g48697, and Bradi3g59300) and investigated their transcription in *Bd* d3_CIM_ izEmb explants with gene-specific probes. Only Bradi3g48697, hereafter referred to as *BdBBMc*, yielded interpretable ISH data (Supplementary Methods). The *BdBBMc* probe strongly labeled cells located in the proliferative bulge, as well as in the coleoptile vasculature, the embryonic root, and some parenchymal cells between the proliferative bulge and the embryonic root (Figure 4e,f). *BdBBMc* gene expression in the scutellum bulges further suggests that these proliferative regions contain embryogenic cells.

In summary, the gene expression patterns described confirmed that tissues in the embryo proper do not undergo drastic developmental shifts following 2,4-D treatment. In that portion of the embryo, cells in developing vascular strands and in apical meristems express a marker of mitotic activity, as well as *WOX* and *BBM* genes that control stem cell homeostasis and pluripotency. In the scutellum, dividing cells that form rapidly growing bulges emerging from a narrow strip of the epithelium express the same genes. The *BdH4*, *BdWOX11*, and *BdBBMc* profiles overlap, but are not identical. They are markedly different from the profile of a control gene, *BdSamDc*, coding for an S-adenosylmethionine decarboxylase component of a polyamine biosynthesis pathway, that is not known to be involved in early embryogenesis or meristem function [45] (Supplementary Figure S3).

Together, our cytological and molecular data suggest that cell clusters with embryogenic potential are established after three days of callogenesis induced by exogenous auxin in d3_CIM_ izEmb explants. However, a functional assay was required to determine whether the proliferative bulges already encompassed functional meristematic regions where secondary somatic embryos could be formed. Therefore, we compared the evolution of d3_CIM_ izEmb explants that were either maintained on CIM (including 2,4-D) or transferred to SIM (including kinetin).

### 2.3. Prolonged Auxin Exposure Only Promotes the Further Proliferation of Established Scutellar Cell Populations

**Figure 5:**
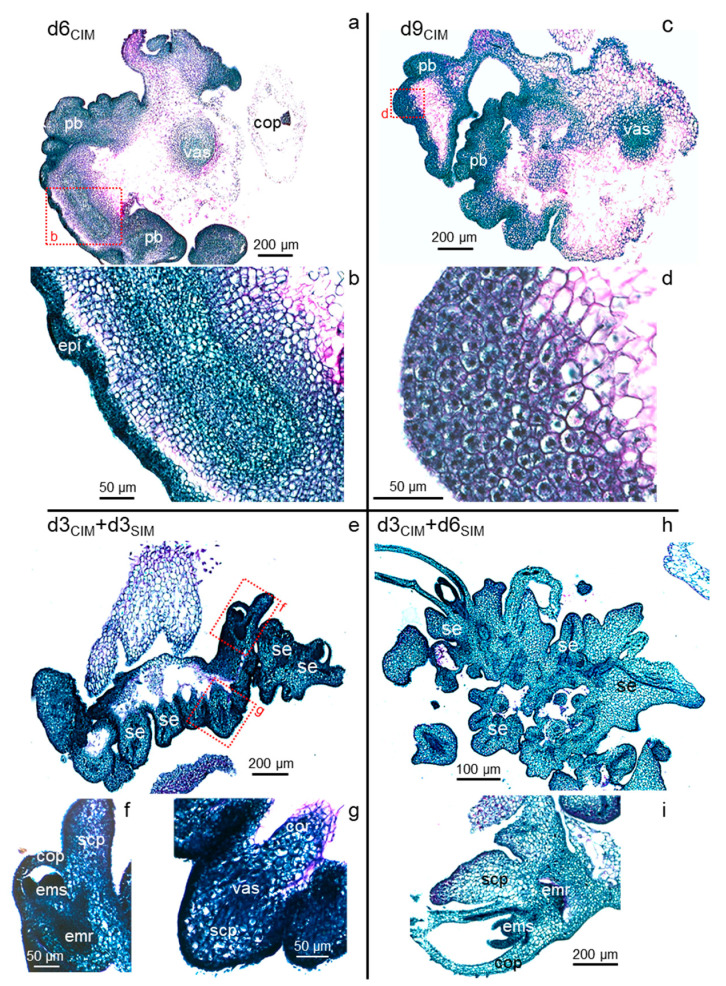
Morphogenesis of secondary somatic embryos. Panels show PAS-NBB-stained cross-sections of izEmb explants, after either continuous treatment with 2,4-D (d6_CIM_ and d9_CIM_, **a**–**d**) or a transfer to kinetin (d3_CIM_ + d3_SIM_ and d3_CIM_ + d6_SIM_, **e**–**i**). Explants maintained on CIM contained proliferating tissues that formed deeply invaginated lobes, in which distinct areas showed different levels of mitotic activity. Non-dividing cells lost their integrity. After transfer to SIM, multiple somatic embryos rapidly emerged in proliferating areas, each characterized by a bipolar axis delimited on either side by the shoot and root apical meristems and flanked by a scutellum. Dotted red boxes indicate close-up views of the cross-section details. cop—coleoptile; cor—coleorhiza; emr—embryonic root; ems—embryonic shoot; epi—scutellum epidermis; pb—proliferative bulge; scp—scutellum parenchyma; se—somatic embryo; vas—scutellum vasculature.

In d6_CIM_ izEmb explants, the proliferative bulges originating from the epidermal and sub-epidermal cell layers had grown in size. The surface of the proliferating zones buckled, yielding secondary bulges with increasingly deep invaginations separating lobes of the explant (Figure 5a). The bulges contained two distinct cell populations: small and very dense peripheral cells, and larger (2 to 3-fold in diameter) inner cells that had thicker cell walls, a lighter stained cytoplasm, and a large—often central—nucleus with one or two nucleoli (Figure 5b). From here on, the initial structure of the zygotic embryo body became more difficult to recognize as the rapid growth of the proliferative areas caused additional tissue bending and tearing. Some explant portions disappeared as very large cells collapsed in the coleorhiza and central scutellum parenchyma, while others disintegrated due to mechanical damage around the alveoli appearing in ruptured tissues.

In d9-10_CIM_ explants, the invaginations expanded further and secondary bulges were readily discernible but remained attached to the primary structure (Figure 5c). Compared to earlier stages, cells in these secondary structures had doubled in size. They contained fragmented vacuoles and a central nucleus surrounded by amyloplasts, and thus appeared clearer and more granular (Figure 5d). These cells also accumulated additional materials in their cell walls that had deep purple coloration and marked junctions with adjacent cells. Together, these morphological changes at the cellular level are typical of the acquisition of the embryogenic fate [6,46]. A fraction of these cells, encased in thickened cell walls, resumed division (Figure 5d). Despite the individualization of such cell clusters and their embryogenic characteristics, SE did not occur in the explants maintained on CIM medium. No bipolar axis, including opposite shoot and root meristems, was ever observed without a transfer to SIM.

### 2.4. Somatic Embryos Can Be Fully Formed within Three Days of Cytokinin Induction and Evolve Rapidly Thereafter 

In contrast, numerous somatic embryos rapidly formed within the proliferating bulge, in the dorsal part of the scutellum, after exposure to kinetin (d3_CIM_ + d3_SIM_; Figure 5e). Ten to fifteen secondary embryos could be seen per explant, but at staggered stages of development. All of them displayed a clearly established bipolar structure within three days of cytokinin treatment, with their root meristem oriented towards the zygotic embryo from which they arose (Figure 5f,g). The newly formed epidermis and vascular tissues were visible in each somatic embryo, as were their incipient scutellum and coleorhiza. All cells in these somatic embryos were dense with a large nucleus. Even though they had developed an outer epidermis and the underlying cell layers of the original zygotic embryo had lost their integrity, the new somatic embryos were not completely detached from the original explant and were still fused together. The d3_CIM_ explants were those tested earliest in a SIM transfer, since the first divisions in the scutellum epidermis were only observed at the d2_CIM_ stage and the resulting cells did not show characteristics associated with pluripotency [6].

The secondary embryos evolved rapidly after initiation and were fully developed after six days of cytokinin treatment (d3_CIM_ + d6_SIM_; Figure 5h). At this stage, both apical meristems were active in each, with small, dark-stained dividing cells (Figure 5i). Their first leaves had already emerged, surrounding the shoot meristem and enclosed in the coleoptile. Reminiscent of the pattern observed in zygotic embryos collected nine days after pollination (Figure 2a), polysaccharides accumulated on the outer surface of the embryonic root, forming a pink, narrow band (Figure 5i). The scutellum had grown in size and included a vascular bundle connected to the embryo proper. The scutellar inner cells were enlarged, with lighter staining. The upper cells, close to the epidermis, accumulated starch (compare Figure 3g and Figure 5i). As observed previously, the embryos remained fused together and attached to the remnants of the initial explant.

Just a few days later (d3_CIM_ + d9_SIM_), individual embryos could be easily and safely separated from the tissues derived from the explant and grown further in vitro in rooting tubes for approximately 2 weeks; they were then transferred into soil where they developed as true-to-type fertile plants. In our hands, the somatic embryos induced within days of tissue culture could not be distinguished from those usually recovered from embryogenic calli maintained for several weeks on CIM.

### 2.5. Brachypodium Zygotic and Somatic Embryogenic Paths Are Very Similar

For further insight into the developmental processes involved, we compared the morphology of the somatic embryos with the features of their zygotic counterparts at successive stages, as described by Hao et al. [47]. Most *Bd* izEmb observed in d3_CIM_ + d3_SIM_ cross-sectioned explants (Figure 5e) corresponded to leaf early (LEE) zygotic embryos, while the remaining minority matched with the leaf middle (LEM) stage. Somatic embryos structured as LEE zygotic embryos were also comparable in size: 200 to 300 µm in length and 150 to 200 µm in width [47] (Figure 1). The polar organization of the forming tissues was obvious in both types, with the upper rounded portion developing into the scutellum and the lower pointed body part into the coleorhiza, while the meristems themselves were not yet visible. In later d3_CIM_ + d6_SIM_ explants, LEM somatic embryos had well-established root and shoot apical meristems, with a growing first leaf. Again, the developmental time was comparable between ZE and SE: in the seed, three days elapsed between the LEE and LEM stages [38], as in the explants analyzed herein (Figure 5e,g).

### 2.6. Taking Advantage of the Precise Timing of Pluripotency Acquisition and Morphogenesis Induction Following the Application of Exogenous Phytohormones

In our system, auxin rapidly triggered division (first observed at d2_CIM_) and a change in cell fate resulting in pluripotent cell clusters that continued proliferating when they were maintained in the same conditions. Full somatic embryos were formed within three days after transfer to an auxin-free medium supplemented with cytokinin. Thus, as classically observed, an initial auxin treatment was indispensable to trigger SE and a subsequent shift to cytokinin was required for secondary embryos to develop.

It is not trivial to determine the precise time and location of the molecular and cellular events that drive cell dedifferentiation and early morphogenesis in complex in vitro cultured explants. As described here, the narrow time windows during which these events take place will facilitate the detailed analysis of plant SE initiation because the investigation of just a few crucial time points may suffice to understand the key regulatory steps involved. Indeed, it is reasonable to assume that the meristematic regions of older calli, in which somatic embryos eventually develop, have the same characteristics as those observed in d3_CIM_ izEmb explants. In other words, morphogenesis in early and late calli most probably follows the same developmental path.

Earlier structures may provide more easily interpretable observations about the relevant cues that drive the resumption of scutellar cell division, the shift to the embryogenic cell fate, and the patterning of the bipolar embryonic axis oriented in tissues that retain their original footprint. For example, in d3_CIM_ + d3_SIM_ explants, the base of all somatic embryos points towards the izEmb scutellum. This orientation of the polar axes potentially results from directed molecular gradients within the explants. It would be difficult to study the mode of action of candidate morphogens in large calli with numerous lobes; however, the relatively simple organization of d3_CIM_ izEmb explants yields itself well to experiments designed for this purpose.

### 2.7. Prospective Applications of Express Somatic Embryogenesis

In most plant regeneration methods, the induction of morphogenesis on media with a higher cytokinin/auxin ratio (or exclusively containing exogenous cytokinin) occurs several weeks after auxin-induced callogenesis. In particular, efficient transformation protocols have been described for B. distachyon, based either on Agrobacterium-mediated gene transfer [42,48] or particle bombardment [49]. The initial steps are the same in all of them: immature zygotic embryos are collected as starting explants to produce transformable embryogenic calli, and a callus prepared for genetic transformation is obtained after six weeks on CIM with two subcultures. Accordingly, the full procedure (from the initiation of tissue culture to the recovery of transgenic plantlets) spans 16 weeks. Our observations show that embryogenic tissues are already present in the scutellum of immature zygotic embryos within three days on 2,4-D medium, and that plantlets can evolve from such explants in only a few additional days (d3_CIM_ + d6_SIM_; Figure 5g). Such a fast track can lead to streamlined transformation protocols that reduce the time and efforts required to generate genetically transformed or gene-edited plants.

## 3. Materials and Methods

### 3.1. Plant Materials

The plant genotype used in this study was the accession Bd21-3 of *B. distachyon* L. [42]. Plants were grown in controlled growth chambers under a 24-h period, with 18 h of light at an intensity of 210 to 280 µmol m^−2^ s^−1^ at plant level. Temperature varied between 22 and 24 °C during the day and was maintained at 20 °C during the night. Relative humidity was kept constant at 60%. Plants grown under these conditions were collected between 14 and 16 days after the emergence of the first inflorescence. Green spikes were sampled, surface sterilized (0.25% active chlorine), and dissected to collect immature zygotic embryos (izEmb) as starting explants for SE induction. izEmbs with a length of 0.4 to 0.6 mm were excised under a binocular in sterile conditions. Explants were cultured on callus-inducing medium (CIM; MS medium supplemented with 0.6 mg/L CuSO_4_, 3% sucrose, 2.5 mg/L 2,4-D, and 0.2% phytagel) and placed in the dark, at 28 °C, for 3 to 10 days. The development of somatic embryos was induced under a 16-h photoperiod at an intensity of 60 μmol m^−^^2^ s^−1^, following the transfer of the explants to shoot-inducing medium (SIM; MS medium supplemented with 3% maltose, 0.2 mg/L kinetin, and 0.2% phytagel) after 3 days (d3_CIM_) or more of culture on CIM.

### 3.2. Histological Analysis

The explants were sampled at daily intervals, starting from day 0 (d0, start of in vitro culture) until d9_CIM_, and at two time points after transfer to SIM (d3_CIM_ + d3_SIM_ and d3_CIM_ + d6_SIM_). Live explants were imaged with a Nikon SMZ800 Stereo Zoom microscope and a Canon PowerShot G11 digital camera. Immature embryos were stained with propidium iodide using a method adapted from Truernit et al. [50] (see Supplementary Methods for details). For all embryo samples analyzed by staining with the periodic acid-Schiff (PAS) reagent, which colors polysaccharides pink, and naphtol blue black (NBB) dye, which colors proteins blue and nuclei dark blue [51], explants were fixed in fresh formaldehyde solution (formaldehyde 4% (Sigma, St. Louis, MO, USA), Triton 1%, and PBS buffer) and incubated overnight at 4 °C. The samples were then gradually dehydrated in an ethanol series (10%, 30%, 50%, 70%, 96%) and incubated overnight in 96% ethanol with 0.1% eosin. After dehydration, each sample was embedded in paraffin as described by Coen et al. [52]. The paraffin blocks were cut in 8 μm cross-sections with an SEC35 low-profile blade on a Leica RM2165 microtome, and subsequently placed on glass slides, on top of water droplets, at 37 °C overnight to promote loosening. After the evaporation of the water, the slides were stored at 4 °C. Cross-sections were PAS-NBB double-stained. The samples were observed and imaged with two microscopes: Zeiss Axioplan2 and Zeiss Axioscan Z2 slide scanner.

### 3.3. RNA in Situ Hybridization 

One μg of total RNA extracted from *Bd* d3_CIM_ izEmb (RNeasy Mini kit, Qiagen, Hilden, Germany) was used as a template to synthesize gene-specific cDNA fragments (Superscript Reverse Transcriptase II kit, Invitrogen, Waltham, MA, USA). Sequence-validated *WUSCHEL-related homeobox 11* (*BdWOX11*), *Histone 4* (*BdH4*), *BABY BOOM* (*BdBBMc*), and *S-adenosylmethionine Decarboxylase* (*BdSamDc*) cDNA fragments were amplified by PCR (Phusion DNA polymerase, Thermo Fisher Scientific, Waltham, MA, USA), gel purified (NucleoSpin Gel and PCR Clean-up Kit, Macherey-Nagel, Düren, Germany), and reverse transcribed in vitro (Riboprobe System kit, Promega, Madison, WI, USA). Additional information is provided on the cDNA probes and their specificity in the Supplementary Materials (Supplementary Table S1; Supplementary Figure S1; Supplementary Methods). Prior to in situ hybridization (ISH), *Bd* d3_CIM_ izEmbs were collected and fixed as described above. Slides with tissue cross-sections were immersed into two successive baths of pure Histo-Clear (10 and 15 min, respectively), then in 100% ethanol (1 min), and subsequently rehydrated in a decreasing ethanol series (100%, 96%, 85% + 0.42% NaCl, 70% + 0.85% NaCl, 50% + 0.85% NaCl, 30% + 0.85% NaCl) for 30 sec each, and finally immersed in 0.85% NaCl for 2 min. Samples were then consecutively transferred to PBS for 2 min, a proteinase K solution (proteinase K (Sigma), 1 mg/L, in Tris 100 mM, pH 7.5; EDTA, 50 mM) for 10 min at 37 °C, a glycine solution (0.2% in PBS) for 2 min, an acetic anhydride/triethanolamine-HCL solution (triethanolamine 1.5% in water, pH 8, HCl adjusted; 0.5% acetic anhydride) for 7 min, and a fresh PBS solution for 2 min. Drops of prehybridization buffer (deionized formamide 50%; 5X SSC; heparin 50 μg/mL; tRNA (Roche, Basel, Switzerland) 100 μg/mL; Tween 0.02%) were deposited on slides and incubation was performed at hybridization temperature for 1 h 30 min. The RNA probes were added to the hybridization buffer (RNA probe 0.4 ng in 10 mL; deionized formamide 50%; dextran sulfate (Sigma) 20%; tRNA (Roche) 1 mg; Tween 0.15%; 100× Denhard; NaCl 0.3 M; Tris 10mM, pH 8; EDTA 1 mM), following denaturation for 2 min at 80 °C, and drops of this solution were pipetted on the hybridization slides. A second slide was placed on top of the first and samples were incubated overnight at hybridization temperature. The slides were then consecutively washed in 0.1× SSC/0.5% SDS (30 min, hybridization T°), 2× SSC/50% formamide (2 h, hybridization T°), NTE (NaCl 2.5 M; Tris 50mM, pH 8; EDTA 5 mM, 5 min, hybridization T°), an RNase solution (RNase (Roche) 10 mg/mL in NTE; 30 min, 37 °C), NTE again (5 min, hybridization T°), formamide 50%/2× SSC (1 h, hybridization T°), 0.1× SSC (2 min, hybridization T°), and finally in PBS overnight at hybridization temperature. For the immunological detection of the hybridized probes, the slides were transferred to blocking buffer (Roche, 0.5%) for 1 h at room temperature, then to wash solution (Tris, 100 mM, pH 7.5; NaCl, 150 mM; BSA [SIGMA] 1%; Triton 100× 0.5%), and then finally immersed with a few drops of the digoxygenin-targeting antibody solution (DIG, Roche, diluted 1/1250 in blocking buffer) and incubated for 1 h at room temperature. The slides were then consecutively washed at room temperature in the same wash solution twice for 20 min, in a second wash solution (Tris, 100 mM, pH 7.5; NaCl 150 mM) for 15 min, and in a third wash solution (Tris 100 mM, pH 9.5; NaCl 100 mM, MgCl_2_ 50 mM) for 15 min. Staining was performed by dipping slides in a staining solution (nitroblue tetrazolium chloride NBT, Roche, 337.5 μg/mL; 5-bromo-4-chloro-3-indolyl-phosphate XP, Roche, 175 μg/mL, in the third washing solution) for 24 h to 48 h in the dark. The reaction was subsequently stopped by incubation in TE at pH 7.5 for 20 min. The slides were rinsed with permuted water, mounted in CoverSafe medium (ref. MMC0226), and stored at 4 °C. RNA ISH samples were observed and imaged with an Axioplan2 microscope (Zeiss).

## 4. Supplementary Methods

### 4.1. Propidium Iodide Staining and Imaging of Bd Immature Embryos

This protocol was adapted from Truernit et al. [50]. *Brachypodium distachyon* immature zygotic embryos were fixed in a 75% ethanol/25% acetic anhydride solution for 4 h at room temperature, then in a 50% ethanol/10% acetic anhydride solution for 2 days. Samples were rehydrated by successive immersion in 50%, 30%, and 10% ethanol, and washed 3 times in distilled water. Amyloplasts were dissolved with amylase (0.2 mg/mL) for 3 h at 37 °C. Fixed explants were washed 3 times in distilled water, incubated in 1% periodic acid for 20 min, rinsed again with water, and stained overnight in Schiff reagent with propidium iodide (PI; 100 mM sodium metabisulphite, 0.15 N HCl, freshly added PI at a final concentration of 0.1 mg/μL). Samples on microscope slides were covered with a chloral hydrate solution (4 g chloral hydrate, 1 mL glycerol, and 2 mL water) after 3 washes in water. Explants were imaged with a Leica SP5 spectral confocal laser scanning microscope (Leica Microsystems, Wetzlar, Germany). The excitation wavelength for PI-stained samples was 488 nm, and the emission signal was collected from 520 to 720 nm.

### 4.2. Characterization of in Situ Hybridization Probes

Histone 4 proteins are encoded in large conserved gene families. In the annotated Bd21-3 genome, ten other genes share at least 90% identity with the fragment of the *H4* gene (BdiBd21-3.1G0918700; Supplementary Table S1) used as an ISH probe in this study. The *BdH4* ISH signal detected is therefore a combination of multiple transcripts; however, it can be interpreted as a single reporter of mitotic patterns since the encoded *H4* proteins are functionally redundant. Within the *WOX* and *BBM* gene families, RNA probes were designed to minimize sequence homology across related members (Supplementary Table S1). In the Bd21-3 genome sequence, the second-best match for the 353-bp probe chosen to detect *BdWOX11* transcripts corresponds to another member of the gene family with which it shares 27.6% identity. The 431-bp probe chosen to detect *BdBBMc* transcripts was also selected to be gene-specific. Dot blot assays reproducing the ISH conditions detected no cross-hybridization between the selected *WOX* and *BBM* probes and other members of their respective gene families (Supplementary Table S2). 

### 4.3. RT-PCR Detection of WOX Gene Expression

Total RNA was extracted from *Bd* izEmbs at different stages of SE induction (d0 to d3_CIM_; three independent biological samples) with an RNeasy Mini kit (Qiagen), including an RNase-Free DNase Set (Qiagen) treatment during washing, according to the manufacturer’s instructions. The Superscript Reverse Transcriptase II kit (Invitrogen) was used to synthesize cDNA from 1 μg of total RNA. DNA was extracted from fresh leaves (Bd21-3) following a protocol adapted from Murray and Thompson [53]. PCR was performed with the DreamTaq DNA Polymerase (Thermo Scientific, Waltham, MA, USA) as follows: 95 °C for 3 min; 30 cycles of 95 °C for 30 s, 55 °C for 30 s, and 72 °C for 1 min 30 s; and finally 72 °C for 7 min. The PCR amplifications of *WUS*/*WOX* gene fragments were performed with gene-specific primers (Supplementary Table S3). The samples analyzed were izEmbs collected from dissected inflorescence nine days after pollination (d0), or one to seven days of in vitro culture on CIM (d1_CIM_ to d7_CIM_). Three *WOX* genes were detected in all three biological replicates: BdiBd21-3.2G0215500 (homologous to Bradi2g16444 in the Bd21 reference genome) detected in izEmb at d0 and d1_CIM_; BdiBd21-3.1G0859400 (Bradi1g63680; *BdWOX11*); and BdiBd21-3.2G0683100 (Bradi2g53390; *BdWOX13a*) detected in izEmb at d0, and from d1_CIM_, to d7_CIM_. The last 2 genes were considered interesting candidates for further characterization because our data suggested that an important switch in cell identity occurred at d3_CIM_.

## Data Availability

All data generated or analyzed during this study are included in this published article and its Supplementary Materials.

## Supplementary Materials

The following supporting information can be downloaded at: https://www.mdpi.com/article/10.3390/plants11081068/s1, Table S1: ISH probe information; Table S2: *Brachypodium distachyon* genes of particular interest in this study; Table S3: *BdWUS/WOX* gene-specific primers; Figure S1: Negative control (sense RNA probes) in situ hybridization; Figure S2: RT-PCR analysis of *Bd WOX* gene expression; Figure S3: Expression of *BdSamDc* in immature zygotic embryos sections.

## Abbreviations

2:4-D—2,4-dichlorophenoxyacetic acid; *Bd*—*Brachypodium distachyon*; CIM—callus-inducing medium; ISH—in situ hybridization; izEmb—immature zygotic embryo; MS medium—Murashige and Skoog medium; SE—somatic embryogenesis; SIM—shoot-inducing medium.
