# Supplementary material for: One-Week Scutellar Somatic Embryogenesis in the Monocot Brachypodium distachyon
Source: Plants (Basel). 2022 Apr 14;11(8):1068. doi: 10.3390/plants11081068 (PMC9025947; doi:10.3390/plants11081068)
Supplement: Supplementary file 1 [file plants-11-01068-s001.zip › Supplementary Figure S1.pptx]

## Slide 1
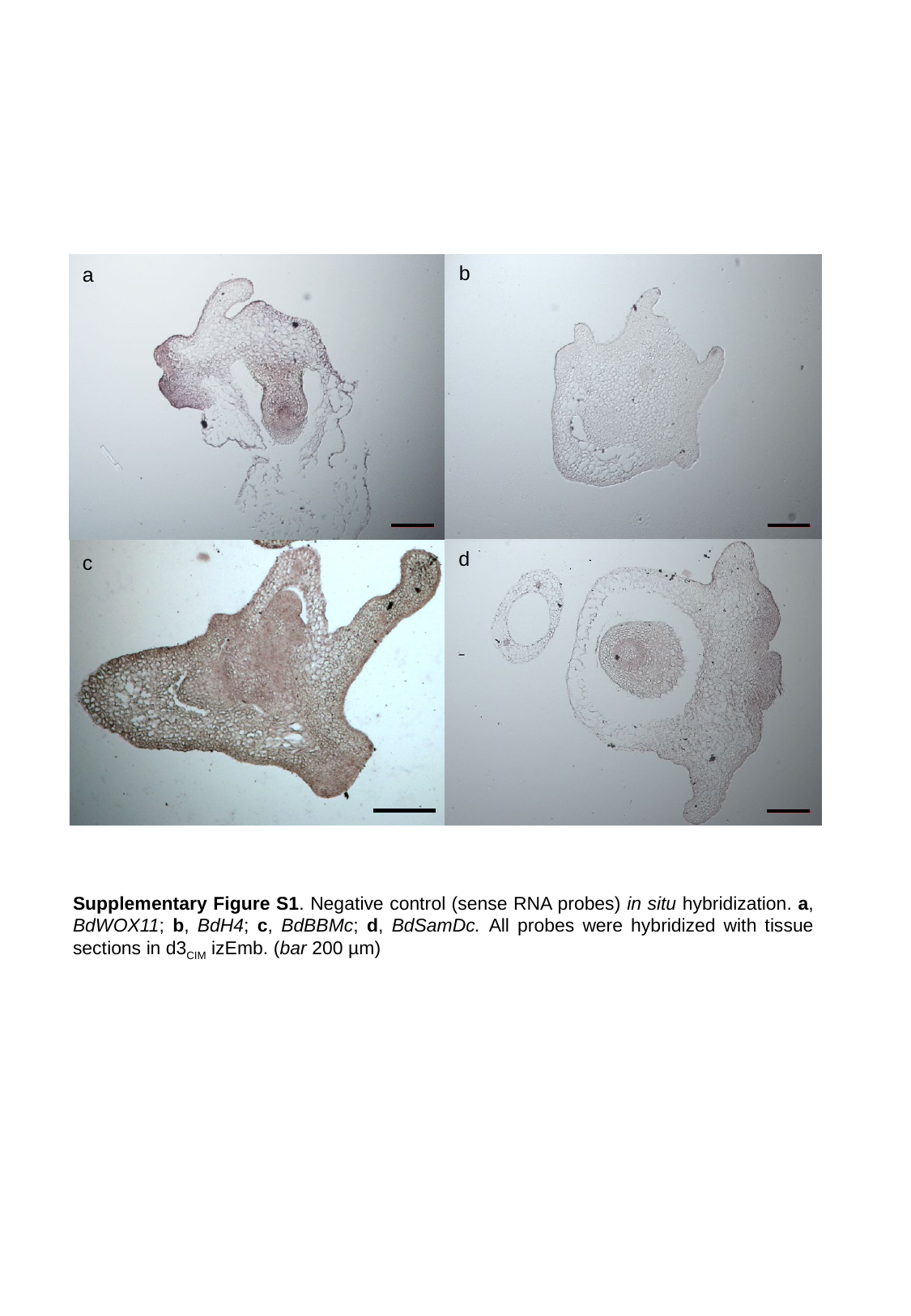

b
a
c
d
Supplementary Figure S1. Negative control (sense RNA probes) in situ hybridization. a, BdWOX11; b, BdH4; c, BdBBMc; d, BdSamDc. All probes were hybridized with tissue sections in d3CIM izEmb. (bar 200 µm)
