# Supplementary material for: One-Week Scutellar Somatic Embryogenesis in the Monocot Brachypodium distachyon
Source: Plants (Basel). 2022 Apr 14;11(8):1068. doi: 10.3390/plants11081068 (PMC9025947; doi:10.3390/plants11081068)
Supplement: Supplementary file 1 [file plants-11-01068-s001.zip › Supplementary Figure S2.pptx]

## Slide 1
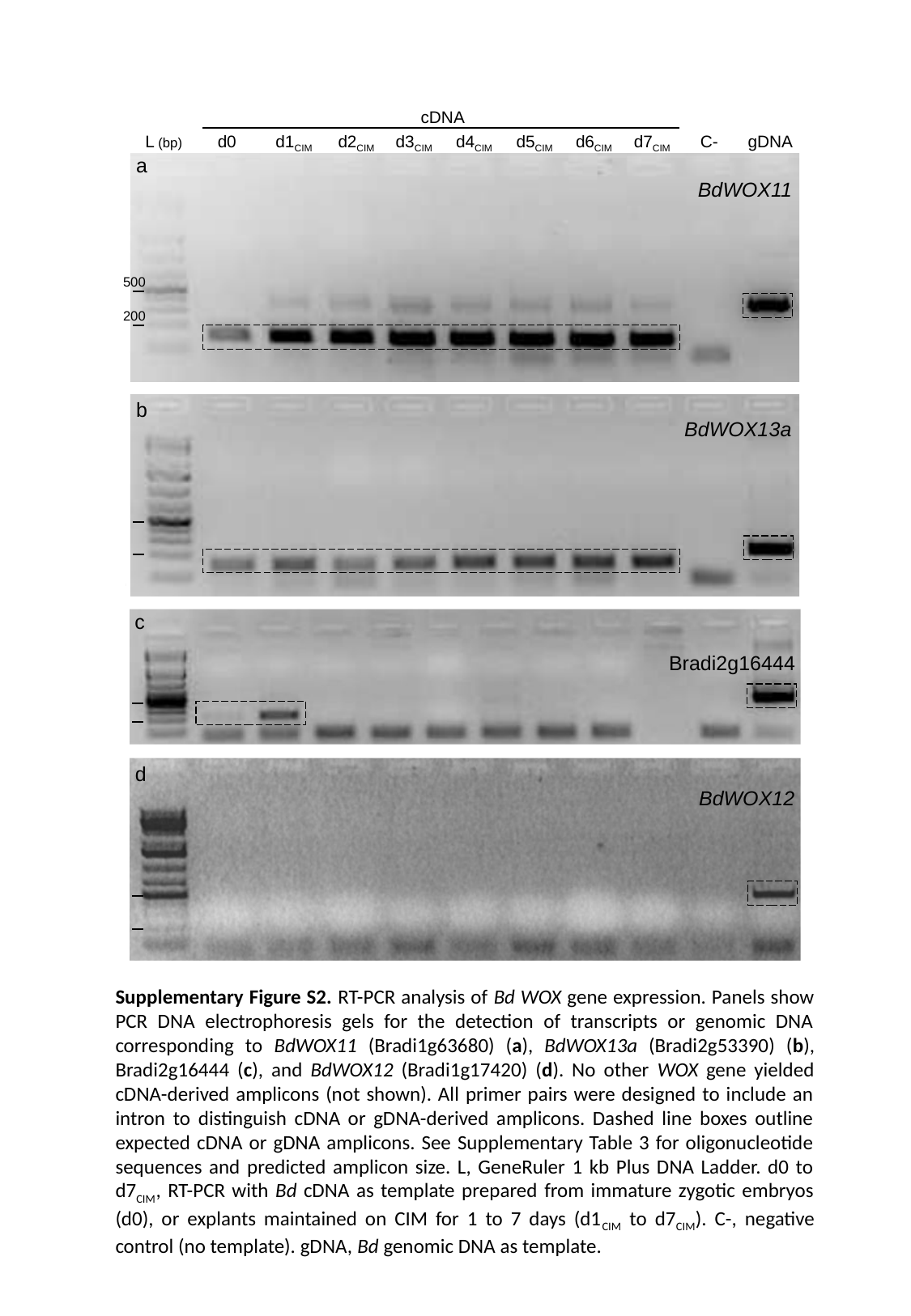

cDNA
d1CIM
d2CIM
d3CIM
d4CIM
d5CIM
d6CIM
d7CIM
gDNA
L (bp)
d0
C-
a
BdWOX11
500
200
b
BdWOX13a
c
Bradi2g16444
d
BdWOX12
Supplementary Figure S2. RT-PCR analysis of Bd WOX gene expression. Panels show PCR DNA electrophoresis gels for the detection of transcripts or genomic DNA corresponding to BdWOX11 (Bradi1g63680) (a), BdWOX13a (Bradi2g53390) (b), Bradi2g16444 (c), and BdWOX12 (Bradi1g17420) (d). No other WOX gene yielded cDNA-derived amplicons (not shown). All primer pairs were designed to include an intron to distinguish cDNA or gDNA-derived amplicons. Dashed line boxes outline expected cDNA or gDNA amplicons. See Supplementary Table 3 for oligonucleotide sequences and predicted amplicon size. L, GeneRuler 1 kb Plus DNA Ladder. d0 to d7CIM, RT-PCR with Bd cDNA as template prepared from immature zygotic embryos (d0), or explants maintained on CIM for 1 to 7 days (d1CIM to d7CIM). C-, negative control (no template). gDNA, Bd genomic DNA as template.
