# Supplementary material for: One-Week Scutellar Somatic Embryogenesis in the Monocot Brachypodium distachyon
Source: Plants (Basel). 2022 Apr 14;11(8):1068. doi: 10.3390/plants11081068 (PMC9025947; doi:10.3390/plants11081068)
Supplement: Supplementary file 1 [file plants-11-01068-s001.zip › Supplementary Figure S3.pptx]

## Slide 1
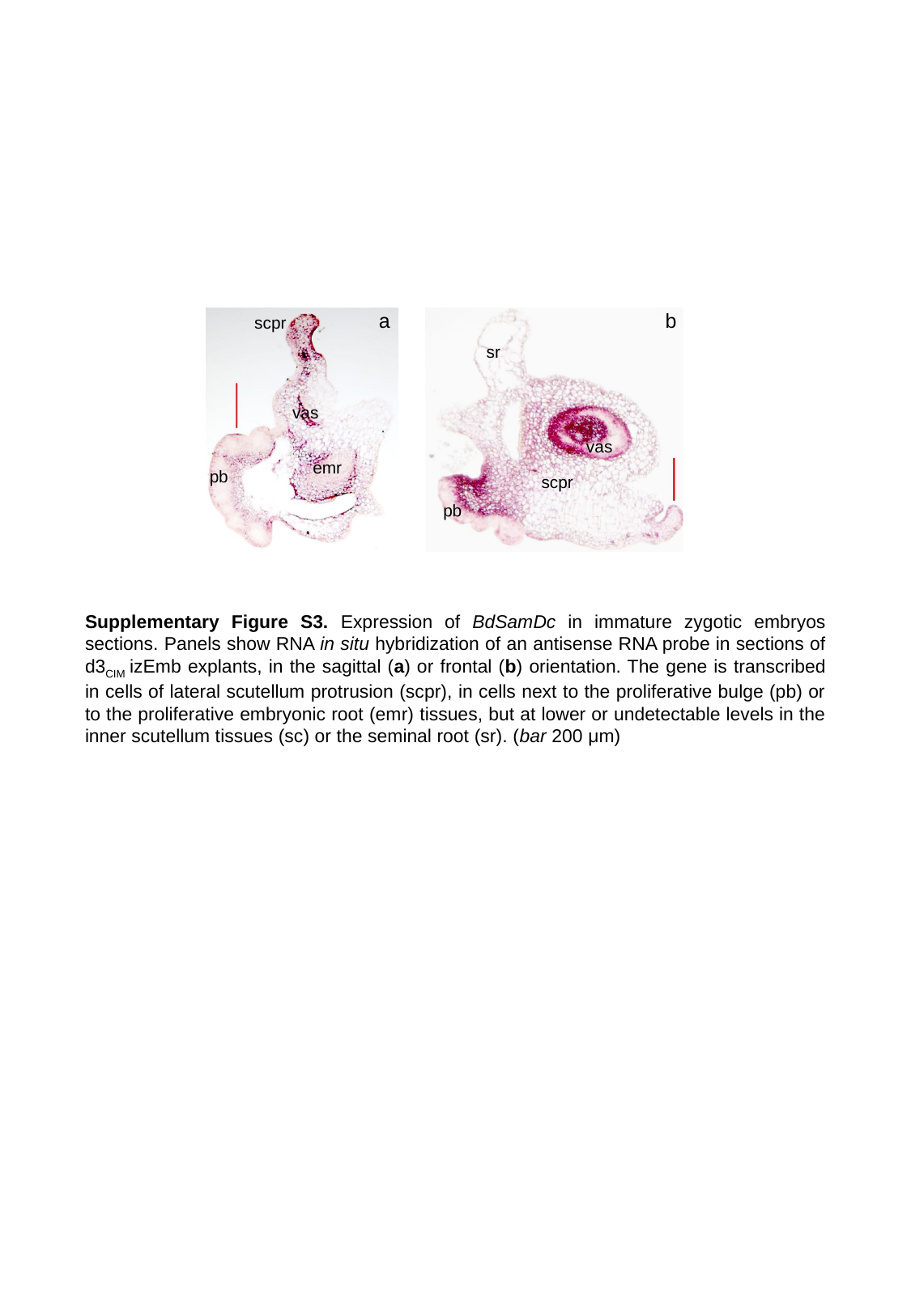

a
scpr
vas
emr
pb
b
sr
vas
scpr
pb
Supplementary Figure S3. Expression of BdSamDc in immature zygotic embryos sections. Panels show RNA in situ hybridization of an antisense RNA probe in sections of d3CIM izEmb explants, in the sagittal (a) or frontal (b) orientation. The gene is transcribed in cells of lateral scutellum protrusion (scpr), in cells next to the proliferative bulge (pb) or to the proliferative embryonic root (emr) tissues, but at lower or undetectable levels in the inner scutellum tissues (sc) or the seminal root (sr). (bar 200 μm)
