# Supplementary material for: One-Week Scutellar Somatic Embryogenesis in the Monocot Brachypodium distachyon
Source: Plants (Basel). 2022 Apr 14;11(8):1068. doi: 10.3390/plants11081068 (PMC9025947; doi:10.3390/plants11081068)
Supplement: Supplementary file 1 [file plants-11-01068-s001.zip › Supplementary Table S1.pdf]

Supplementary Table S1. ISH probe information

Gene	Probe	Primer sequences (5' > 3') for cDNA PCR amplification and revere transcription (T7 promoter in bold)	Length (bp)	Hybridization temperature (°C)
Bradi1g63680 *WOX11*	Sense	FW-**T7**: **TGTAATACGACTCACTATAGGGC**TGCCGCCAACAATAACAATACCAG	353	53.8
		RV: TCGAGATGGCGAAGAGATCGTC		
	Antisense	FW: TGCCGCCAACAATAACAATACCAG		
		RV-**T7**: **TGTAATACGACTCACTATAGGGC**TCGAGATGGCGAAGAGATCGTC		
Bradi1g68190 *H4*	Sense	FW-**T7**: **TGTAATACGACTCACTATAGGGC**AAGCGCCACAGGAAGGTTC	313	51.5
		RV: CGACACAGACAGGTACTCTCGG		
	Antisense	FW: AAGCGCCACAGGAAGGTTC		
		RV-**T7**: **TGTAATACGACTCACTATAGGGC**CGACACAGACAGGTACTCTCGG		
Bradi3g48697 *BBMc*	Sense	FW-**T7**: **TGTAATACGACTCACTATAGGGC**AGCCTCACCAGGATAGCTCG	431	52.0
		RV: CATGGACAGCTCCATGGTGT		
	Antisense	FW: AGCCTCACCAGGATAGCTCG		
		RV-**T7**: **TGTAATACGACTCACTATAGGGC**CATGGACAGCTCCATGGTGT		
Bradi5g14640 *SamDc*	Sense	FW-**T7: TGTAATACGACTCACTATAGGGC**GTTCATCTTCCCTGGCGCAC	712	48.0
		RV: GCAGTGGAACACCGATCTCG		
	Antisense	FW: GTTCATCTTCCCTGGCGCAC		
		RV-**T7: TGTAATACGACTCACTATAGGGCGC**AGTGGAACACCGATCTCG		

Probe hybridization temperature = melting temperature (Tm) - 15°C
Tm = 81.5 + 16.6 Log [Na+] + 0.41 x % (G+C content) - 0.61 x % Formamide - (500/N), where [Na+] = Sodium concentration = 0.66 M; % Formamide = 60; N= probe length (in bp); % (C+G) = (number of C + number of G)/ N * 100.
