# Supplementary material for: One-Week Scutellar Somatic Embryogenesis in the Monocot Brachypodium distachyon
Source: Plants (Basel). 2022 Apr 14;11(8):1068. doi: 10.3390/plants11081068 (PMC9025947; doi:10.3390/plants11081068)
Supplement: Supplementary file 1 [file plants-11-01068-s001.zip › Supplementary Table S2.pdf]

Supplementary Table S2. *Brachypodium distachyon* genes of particular interest in this study

Gene name	Family name	Gene ID in Bd21	Gene ID in Bd21-3
BBMa	*BBM*	Bradi1g64240	BdiBd21-3.1G0866800
BBMb	*BBM*	Bradi2g57747	BdiBd21-3.2G0739100
BBMc	*BBM*	Bradi3g48697	BdiBd21-3.3G0645400
BBMd	*BBM*	Bradi3g59300	BdiBd21-3.3G0781000
BdH4	*H4*	Bradi4g68190	BdiBd21-3.1G0918700
SamDc	*SamDC*	Bradi5g14640	BdiBd21-3.5G0187900
BdWOX12	*WOX*	Bradi1g17420	BdiBd21-3.1G0227200
BdWOX11	*WOX*	Bradi1g63680	BdiBd21-3.1G0859400
BdWOX13b	*WOX*	Bradi1g69185	BdiBd21-3.1G0933100
	WOX	Bradi2g16444	BdiBd21-3.2G0215500
BdWOX3	*WOX*	Bradi2g37650	BdiBd21-3.2G0477300
BdWOX9	*WOX*	Bradi2g46055	BdiBd21-3.2G0587500
BdWOX13a	*WOX*	Bradi2g53390	BdiBd21-3.2G0683100
BdWOX2	*WOX*	Bradi2g54590	BdiBd21-3.2G0698100
BdWOX5	*WOX*	Bradi2g55270	BdiBd21-3.2G0707000
	WOX	Bradi3g18800	BdiBd21-3.3G0261200
BdNS1	*WOX*	Bradi4g45325	BdiBd21-3.4G0627800
BdWOX4	*WOX*	Bradi5g24080	BdiBd21-3.5G0316500
BdWUSCHEL	*WOX*	Bradi5g25113	BdiBd21-3.5G0330100
