# Supplementary material for: One-Week Scutellar Somatic Embryogenesis in the Monocot Brachypodium distachyon
Source: Plants (Basel). 2022 Apr 14;11(8):1068. doi: 10.3390/plants11081068 (PMC9025947; doi:10.3390/plants11081068)
Supplement: Supplementary file 1 [file plants-11-01068-s001.zip › Supplementary Table S3.pdf]

Supplementary Table S3. *BdWUS/WOX* gene-specific primers

WUS/WOX genes	Template	Product length (bp)	Forward primer sequences (5' > 3')	Reverse primer sequences (5' > 3')
Bradi1g17420 *BdWOX12*	gDNA	470	TGCCATCTCGAGGCAGATGG	TTCTTCCGTAGCAACGCACG
	cDNA	337	CCAGGTGTACATCAATGGCG	TTCTTCCGTAGCAACGCACG
Bradi1g63680 *BdWOX11*	gDNA	360	CCTCATTCAGAGCCTGCAAATG	CCCGGACACCCCATACATATC
	cDNA	139	CCTCATTCAGAGCCTGCAAATG	CCCGGACACCCCATACATATC
Bradi1g69185 *BdWOX13b*	gDNA	1448	GGTCATGACTGACGAGCAGATG	CCATGAACCATCAGAGGATCGC
	cDNA	165	GGTCATGACTGACGAGCAGATG	CCATGAACCATCAGAGGATCGC
Bradi2g16444	gDNA	688	GGCTTCGAGCAGTACCAGCC	AGTTGTTAGAGCCGAGCACG
	cDNA	451	GGCTTCGAGCAGTACCAGCC	AGTTGTTAGAGCCGAGCACG
Bradi2g37650 *BdWOX3*	gDNA	400	GCGTCTCTCCGGTGCC	TCGTCCTTGACGGAGGCTCC
	cDNA	265	GCGTCTCTCCGGTGCC	TCGTCCTTGACGGAGGCTCC
Bradi2g46055 *BdWOX9*	gDNA	679	TCGCCGAACAGGCAGACTTG	AACTGCTGCTGTTTGGGCTG
	cDNA	488	TCGCCGAACAGGCAGACTTG	AACTGCTGCTGTTTGGGCTG
Bradi2g53390 *BdWOX13a*	gDNA	221	GGGCCAGATGTCCTTCTATGC	CGCCGTGTCATCCATACATG
	cDNA	134	GGGCCAGATGTCCTTCTATGC	CGCCGTGTCATCCATACATG
Bradi2g54590 *BdWOX2*	gDNA	1895	GGCTCCTGTTCCTCTTCTTCCTG	AAGTAGTGGGCTGCAGTTGC
	cDNA	120	GGCTCCTGTTCCTCTTCTTCCTG	AAGTAGTGGGCTGCAGTTGC
Bradi2g55270 *BdWOX5*	gDNA	250	GCCCGCGAGGACCT	CCCTCGTCGGCCG
	cDNA	148	GCCCGCGAGGACCT	CCCTCGTCGGCCG
Bradi3g18800	gDNA	268	CGCCATCCCTCGGC	CGTCGTGCCCAAACGTG
	cDNA	161	CGCCATCCCTCGGC	CGTCGTGCCCAAACGTG
Bradi4g45325 *BdNS1*	gDNA	246	CCGTGATGCAGCTTCACCAT	CCAGCTGCTGGAAACTCCAAC
	cDNA	151	CCGTGATGCAGCTTCACCAT	CCAGCTGCTGGAAACTCCAAC
Bradi5g24080 *BdWOX4*	gDNA	545	CTCTTCCTCTGCGTTGTCGC	GGGAATCGGAAGAGTCGTCG
	cDNA	437	CTCTTCCTCTGCGTTGTCGC	GGGAATCGGAAGAGTCGTCG
Bradi5g25113 *BdWUSCHEL*	gDNA	430	CTCGGCTCATCTGCAGATGG	CGATTTCTTGGTGGCCTCCG
	cDNA	272	CTCGGCTCATCTGCAGATGG	CGATTTCTTGGTGGCCTCCG
